# Supplementary material for: Field evaluation of drone and AI assisted larval source management in Ghana
Source: PLoS One. 2026 Feb 4;21(2):e0340690. doi: 10.1371/journal.pone.0340690 (PMC12872003; doi:10.1371/journal.pone.0340690)
Supplement: Attachment — Submitted filename: Response_to_Reviewers_auresp_2.docx [file pone.0340690.s003.docx]

**Manuscript ID: PONE-D-25-27483**

**Title: Field evaluation of drone and AI assisted larval source management in Ghana**

**・General Response**

Dear Editor and Reviewers,

We sincerely thank you for your constructive comments and valuable suggestions on our manuscript. We have carefully revised the manuscript in response to all the points raised. Below, we provide a detailed point-by-point response. Reviewer comments are presented in **bold italics**, followed by our responses. All changes in the revised manuscript are indicated using track changes.

**・Response to Journal Requirements:**

***1 When submitting your revision, we need you to address these additional requirements. Please ensure that your manuscript meets PLOS ONE's style requirements, including those for file naming.***

**Response:**

We have revised the manuscript and files to comply with PLOS ONE’s style and formatting requirements, including the file naming conventions.

***2 Please include a complete copy of PLOS’ questionnaire on inclusivity in global research in your revised manuscript.***

**Response:**

As instructed, we have completed the questionnaire on inclusivity in global research and included it as Supporting Information in the revised submission.

***3 Please note that PLOS ONE has specific guidelines on code sharing for submissions in which author-generated code underpins the findings in the manuscript. In these cases, we expect all author-generated code to be made available without restrictions upon publication of the work. Please review our guidelines at https://journals.plos.org/plosone/s/materials-and-software-sharing#loc-sharing-code and ensure that your code is shared in a way that follows best practice and facilitates reproducibility and reuse.***

**Response:**

We acknowledge PLOS ONE’s policy regarding code sharing. In our revised manuscript, we have provided a detailed description of the AI methodology at a level sufficient to allow reproducibility of the study’s findings. However, we respectfully note that the underlying software implementation, including the trained AI models and author-generated scripts, forms part of proprietary assets developed by our company. To protect commercial interests, we are unable to make the raw code publicly available.

We believe that the methodological detail provided in the manuscript ensures transparency and scientific reproducibility, while the restriction on code release is necessary to safeguard intellectual property. We kindly request the editor’s consideration of this exemption in line with PLOS ONE’s data and materials policy.

***4 Thank you for stating in your Funding Statement:***

***This study was supported by funding from the Japan International Cooperation Agency (JICA) and the Japan Science and Technology Agency (JST) under the framework of international technical cooperation. The funding was administered through SORA Technology Ltd., which coordinated the implementation activities. The funders had no direct role in the decision to submit the manuscript for publication.***

***Please provide an amended statement that declares *all* the funding or sources of support (whether external or internal to your organization) received during this study, as detailed online in our guide for authors at http://journals.plos.org/plosone/s/submit-now. Please also include the statement “There was no additional external funding received for this study.” in your updated Funding Statement.***

***Please include your amended Funding Statement within your cover letter. We will change the online submission form on your behalf.***

**Response:**

As instructed, we have amended the Funding Statement to declare all sources of support and have included the required statement regarding additional external funding. The revised Funding Statement has been included in the cover letter.

***5 Thank you for stating the following in the Competing Interests section:***

***This study was conducted with funding administered through SORA Technology Ltd., to which authors Masaki Umeda and Juhoe Kim are affiliated. Authors Godfred A. Bokpin, and Francis A. Adzei received research support through this funding mechanism. Authors affiliated with SORA Technology were involved in the study design, implementation, and manuscript preparation. All other authors declare no competing interests.***

***We note that one or more of the authors are employed by a commercial company.***

***a. Please provide an amended Funding Statement declaring this commercial affiliation, as well as a statement regarding the Role of Funders in your study. If the funding organization did not play a role in the study design, data collection and analysis, decision to publish, or preparation of the manuscript and only provided financial support in the form of authors' salaries and/or research materials, please review your statements relating to the author contributions, and ensure you have specifically and accurately indicated the role(s) that these authors had in your study. You can update author roles in the Author Contributions section of the online submission form.***

***Please also include the following statement within your amended Funding Statement.***

***“The funder provided support in the form of salaries for authors [insert relevant initials], but did not have any additional role in the study design, data collection and analysis, decision to publish, or preparation of the manuscript. The specific roles of these authors are articulated in the ‘author contributions’ section.”***

***If your commercial affiliation did play a role in your study, please state and explain this role within your updated Funding Statement.***

***b. Please also provide an updated Competing Interests Statement declaring this commercial affiliation along with any other relevant declarations relating to employment, consultancy, patents, products in development, or marketed products, etc.***

***Within your Competing Interests Statement, please confirm that this commercial affiliation does not alter your adherence to all PLOS ONE policies on sharing data and materials by including the following statement: "This does not alter our adherence to PLOS ONE policies on sharing data and materials.” (as detailed online in our guide for authors http://journals.plos.org/plosone/s/competing-interests) . If this adherence statement is not accurate and there are restrictions on sharing of data and/or materials, please state these. Please note that we cannot proceed with consideration of your article until this information has been declared.***

***Please include both an updated Funding Statement and Competing Interests Statement in your cover letter. We will change the online submission form on your behalf.***

**Response:**

As instructed, we have revised both the Funding Statement and the Competing Interests Statement to clearly declare the commercial affiliation with SORA Technology and to follow the required format. The amended versions have been included in the cover letter.

***6 We note that your Data Availability Statement is currently as follows: All relevant data are within the manuscript and its Supporting Information files.***

***Please confirm at this time whether or not your submission contains all raw data required to replicate the results of your study. Authors must share the “minimal data set” for their submission. PLOS defines the minimal data set to consist of the data required to replicate all study findings reported in the article, as well as related metadata and methods (https://journals.plos.org/plosone/s/data-availability#loc-minimal-data-set-definition).***

***For example, authors should submit the following data:***

***- The values behind the means, standard deviations and other measures reported;***

***- The values used to build graphs;***

***- The points extracted from images for analysis.***

***Authors do not need to submit their entire data set if only a portion of the data was used in the reported study.***

***If your submission does not contain these data, please either upload them as Supporting Information files or deposit them to a stable, public repository and provide us with the relevant URLs, DOIs, or accession numbers. For a list of recommended repositories, please see https://journals.plos.org/plosone/s/recommended-repositories.***

***If there are ethical or legal restrictions on sharing a de-identified data set, please explain them in detail (e.g., data contain potentially sensitive information, data are owned by a third-party organization, etc.) and who has imposed them (e.g., an ethics committee). Please also provide contact information for a data access committee, ethics committee, or other institutional body to which data requests may be sent. If data are owned by a third party, please indicate how others may request data access.***

**Response:**

We acknowledge that the data underlying Figures 4, 5, 7, 8, 9, and 10 are required to allow replication of our results. Accordingly, we have prepared the relevant datasets and submitted them as CSV files under Supporting Information through the PLOS ONE submission system.

***7 When completing the data availability statement of the submission form, you indicated that you will make your data available on acceptance. We strongly recommend all authors decide on a data sharing plan before acceptance, as the process can be lengthy and hold up publication timelines. Please note that, though access restrictions are acceptable now, your entire data will need to be made freely accessible if your manuscript is accepted for publication. This policy applies to all data except where public deposition would breach compliance with the protocol approved by your research ethics board. If you are unable to adhere to our open data policy, please kindly revise your statement to explain your reasoning and we will seek the editor's input on an exemption. Please be assured that, once you have provided your new statement, the assessment of your exemption will not hold up the peer review process.***

**Response:**

We acknowledge the importance of ensuring timely data availability. In line with PLOS ONE’s open data policy, we have already prepared and submitted the minimal dataset required to replicate our findings. Specifically, the data underlying Figures 4, 5, 7, 8, 9, and 10 have been provided as CSV files and uploaded as Supporting Information through the submission system. These files will therefore be freely accessible upon publication.

***8 Please include your full ethics statement in the ‘Methods’ section of your manuscript file. In your statement, please include the full name of the IRB or ethics committee who approved or waived your study, as well as whether or not you obtained informed written or verbal consent. If consent was waived for your study, please include this information in your statement as well.***

**Response:**

As instructed, we have included a full Ethics Statement in the Methods section of the revised manuscript. The statement now specifies the approving institutions (the National Malaria Elimination Program and the Ministry of Health of Ghana), the process of obtaining informed consent from community representatives, and confirmation that no deviations from the approved study protocol occurred.

***9 We note that Figures 2 and 3 in your submission contain map/satellite images which may be copyrighted. All PLOS content is published under the Creative Commons Attribution License (CC BY 4.0), which means that the manuscript, images, and Supporting Information files will be freely available online, and any third party is permitted to access, download, copy, distribute, and use these materials in any way, even commercially, with proper attribution. For these reasons, we cannot publish previously copyrighted maps or satellite images created using proprietary data, such as Google software (Google Maps, Street View, and Earth). For more information, see our copyright guidelines: http://journals.plos.org/plosone/s/licenses-and-copyright.***

***We require you to either present written permission from the copyright holder to publish these figures specifically under the CC BY 4.0 license, or remove the figures from your submission:***

***a. You may seek permission from the original copyright holder of Figures 2 and 3 to publish the content specifically under the CC BY 4.0 license.***

***We recommend that you contact the original copyright holder with the Content Permission Form (http://journals.plos.org/plosone/s/file?id=7c09/content-permission-form.pdf) and the following text:***

***“I request permission for the open-access journal PLOS ONE to publish XXX under the Creative Commons Attribution License (CCAL) CC BY 4.0 (http://creativecommons.org/licenses/by/4.0/). Please be aware that this license allows unrestricted use and distribution, even commercially, by third parties. Please reply and provide explicit written permission to publish XXX under a CC BY license and complete the attached form.”***

***Please upload the completed Content Permission Form or other proof of granted permissions as an "Other" file with your submission.***

***In the figure caption of the copyrighted figure, please include the following text: “Reprinted from [ref] under a CC BY license, with permission from [name of publisher], original copyright [original copyright year].”***

***b. If you are unable to obtain permission from the original copyright holder to publish these figures under the CC BY 4.0 license or if the copyright holder’s requirements are incompatible with the CC BY 4.0 license, please either i) remove the figure or ii) supply a replacement figure that complies with the CC BY 4.0 license. Please check copyright information on all replacement figures and update the figure caption with source information. If applicable, please specify in the figure caption text when a figure is similar but not identical to the original image and is therefore for illustrative purposes only.***

***The following resources for replacing copyrighted map figures may be helpful:***

***USGS National Map Viewer (public domain): http://viewer.nationalmap.gov/viewer/***

***The Gateway to Astronaut Photography of Earth (public domain): http://eol.jsc.nasa.gov/sseop/clickmap/***

***Maps at the CIA (public domain): https://www.cia.gov/library/publications/the-world-factbook/index.html and https://www.cia.gov/library/publications/cia-maps-publications/index.html***

***NASA Earth Observatory (public domain): http://earthobservatory.nasa.gov/***

***Landsat: http://landsat.visibleearth.nasa.gov/***

***USGS EROS (Earth Resources Observatory and Science (EROS) Center) (public domain): http://eros.usgs.gov/#***

***Natural Earth (public domain): http://www.naturalearthdata.com/***

**Response:**

We appreciate the editor’s concern regarding the use of copyrighted map/satellite images. In response, we have replaced Figures 2 and 3 with maps generated from OpenStreetMap, which is distributed under the Open Database License (ODbL) with map tiles licensed under CC BY-SA 2.0. We have revised the figure captions to include appropriate attribution (“Background map tiles © OpenStreetMap contributors, licensed under CC BY-SA 2.0. Additional annotations by the authors.”). No proprietary map sources (e.g., Google Maps or Google Earth) were used in the revised figures.

***10 We note that Figure 1 in your submission contain copyrighted images. All PLOS content is published under the Creative Commons Attribution License (CC BY 4.0), which means that the manuscript, images, and Supporting Information files will be freely available online, and any third party is permitted to access, download, copy, distribute, and use these materials in any way, even commercially, with proper attribution. For more information, see our copyright guidelines: http://journals.plos.org/plosone/s/licenses-and-copyright.***

***We require you to either present written permission from the copyright holder to publish these figures specifically under the CC BY 4.0 license, or remove the figures from your submission:***

***a. You may seek permission from the original copyright holder of Figure 1 to publish the content specifically under the CC BY 4.0 license.***

***We recommend that you contact the original copyright holder with the Content Permission Form (http://journals.plos.org/plosone/s/file?id=7c09/content-permission-form.pdf) and the following text:***

***“I request permission for the open-access journal PLOS ONE to publish XXX under the Creative Commons Attribution License (CCAL) CC BY 4.0 (http://creativecommons.org/licenses/by/4.0/). Please be aware that this license allows unrestricted use and distribution, even commercially, by third parties. Please reply and provide explicit written permission to publish XXX under a CC BY license and complete the attached form.”***

***Please upload the completed Content Permission Form or other proof of granted permissions as an "Other" file with your submission.***

***In the figure caption of the copyrighted figure, please include the following text: “Reprinted from [ref] under a CC BY license, with permission from [name of publisher], original copyright [original copyright year].”***

***b. If you are unable to obtain permission from the original copyright holder to publish these figures under the CC BY 4.0 license or if the copyright holder’s requirements are incompatible with the CC BY 4.0 license, please either i) remove the figure or ii) supply a replacement figure that complies with the CC BY 4.0 license. Please check copyright information on all replacement figures and update the figure caption with source information. If applicable, please specify in the figure caption text when a figure is similar but not identical to the original image and is therefore for illustrative purposes only.***

**Response:**

We appreciate the editor’s concern regarding the use of copyrighted map/satellite images. In response, we have replaced Figures 1 with maps generated from OpenStreetMap, which is distributed under the Open Database License (ODbL) with map tiles licensed under CC BY-SA 2.0. We have revised the figure captions to include appropriate attribution (“Background map tiles © OpenStreetMap contributors, licensed under CC BY-SA 2.0. Additional annotations by the authors.”). No proprietary map sources (e.g., Google Maps or Google Earth) were used in the revised figures.

***11 If the reviewer comments include a recommendation to cite specific previously published works, please review and evaluate these publications to determine whether they are relevant and should be cited. There is no requirement to cite these works unless the editor has indicated otherwise.***

**Response:**

Thank you for the clarification regarding citations. The reviewers did not request us to cite any specific publications. However, they suggested adding some reference to related areas. We have accordingly revised the manuscript to include a brief mention of this field, so that the context of our study is more clearly situated within the broader literature.

**・Response to Reviewer #1:**

***1 As stated by authors, the detection of waterbodies was still conducted manually using drone images, with only minimal support from image recognition AI. Then I did not find any novelty in the manuscript. Until we are not detecting the breeding sites with automated AI enabled drone technology, it is not of much use. Further, drone images did not identify the sites positive for larvae. Even limited success in the same might have resulted in a lot of output.***

**Response:**

We respectfully disagree with the reviewer’s concern. While previous studies have explored automated detection of waterbodies using image recognition AI, such methods are not yet standardized nor widely reliable. In this study, we focused on advancing beyond simple habitat detection by integrating AI-based risk classification into a three-step pipeline of drone imagery, waterbody identification, and operational deployment. Importantly, image recognition AI relies on human-annotated training data, meaning its performance is unlikely to surpass that of skilled human annotators; therefore, replacing this step with manual identification does not compromise the validity of our approach. The novelty of our work lies in demonstrating, for the first time, the operational value of combining drone mapping with AI-based risk prioritization, which significantly improved the efficiency of larval source management. This implementation-focused evaluation provides an important and practical contribution to the field. To clarify this point, we have revised the Method section to explicitly describe the manual identification step and expanded the Discussion to explain the rationale for this choice and to highlight the novelty of integrating risk classification AI into operational workflows.

***2 The AI model section lacks methodological details, particularly regarding model validation and feature importance. How the CatBoost model was trained and validated (e.g., dataset split, hyperparameter tuning). Also provide limitations of the model’s precision and implications for potential overtreatment.***

**Response:**

Thank you for this important comment. The Methods section has been revised to provide additional methodological details. We now describe the training procedure of the CatBoost model, including the fixed decision threshold, early stopping, and maximum tree depth, as well as the stratified 5-fold cross-validation used for internal validation (80% training / 20% testing per fold with class balance preserved).

In addition, we have included the results of feature importance analysis derived from the trained CatBoost model. Normalized relative feature importance (RFI) values are now presented in Table 2, showing that waterbody type and visual turbidity were comparatively more influential predictors, while other features contributed at moderate or lower levels.

Finally, we have clarified the operational interpretation of model performance metrics. Precision influences overtreatment: higher precision reduces unnecessary treatment of low-risk waterbodies, while lower precision increases overtreatment but remains comparable to existing operations that apply larvicides to all detected waterbodies. By contrast, recall governs the likelihood of missing high-risk habitats, which would have more severe public-health consequences. For this reason, recall was prioritized in model design, with improvements in precision pursued only insofar as they do not compromise recall.

***3 Use of 100 m height for drone application for time efficiency is not justified. As better resolution of images at 50 m height might have provided good results in larval site identification. Please clarify in detail.***

**Response:**

We appreciate the reviewer’s valuable comment. The rationale for selecting 100 m altitude has been described in the Methods section, and we have further revised the text to make this point clearer. Specifically, test flights were conducted at both 50 m and 100 m to evaluate the trade-off between resolution and efficiency. While 50 m flights provided higher resolution, they required about 20 minutes per grid compared to only 5 minutes at 100 m. At 100 m, the images were still sufficient to detect surface waterbodies several tens of centimeters in diameter, with a ground sampling distance of approximately 2.6 cm/pixel. In addition, operational safety considerations such as terrain variations within sub-districts and the risk of collisions with tall structures like transmission towers provided further justification for adopting 100 m. Therefore, an altitude of 100 m was adopted as the standard flight height to balance adequate image quality, time efficiency, and operational safety. The Methods section has been revised accordingly to clarify this rationale.

***4 There are no details of larvicide used in the manuscript. Kindly provide the same.***

**Response:**

Thank you for this comment. We have revised the Methods section in line xx to xx to clarify the larvicide used in our study. Both the conventional and proposed LSM approaches employed VectoLex WDG, a biological larvicide containing Bacillus sphaericus 2362 (strain ABTS-1743), with a reported residual efficacy of 3–4 weeks. VectoLex is formulated as a water-dispersible granule and can be applied with standard ground or aerial equipment across diverse aquatic habitats. In Ghana, larvicide selection and application protocols are determined by the National Malaria Elimination Program (NMEP), under which VectoLex has been adopted as the standard product for operational LSM activities. Our study therefore followed existing national policy, with the primary focus placed on evaluating the feasibility of drone- and AI-assisted mapping rather than reassessing the baseline efficacy of the larvicide.

***5 How the larval risk criteria were defined? Kindly provide.***

**Response:**

We thank the reviewer for pointing out the need for clarification. The larval risk was defined as a binary variable based on the presence of mosquito larvae in waterbodies: sites with at least one larva were classified as high risk 1, while sites without larvae were classified as low risk 0. The AI model was trained as a binary classifier to predict this risk, outputting probabilities between 0 and 1, which were then thresholded at 0.5 to assign the final class. We have added this description to the Method section for clarity.

***6 Kindly provide details of how many habitats came under high risk larval sites. It will justify the claims of reduction in larvicide usage. Further, show the difference in area of breeding habitat sprayed both under conventional and targeted larviciding.***

**Response:**

We thank the reviewer for this important comment. The number of habitats classified as high-risk by the AI model and subsequently sprayed has been presented both in the main text and in Table 7, where “sprayed sites” correspond to high-risk habitats. For example, in Nkwantanan, 73 out of 131 detected habitats were classified as high-risk and treated. These ratios (0.557 in Nkwantanan, 0.592 in Abaam, 0.747 in Takyiman, and 0.585 in Asuom) directly indicate the proportion of habitats prioritized for treatment compared with the control arm, in which all detected habitats were sprayed without risk classification.

Regarding the comparison of treated area, we acknowledge the reviewer’s point. Although some information on habitat size was recorded during field operations, it was not done in a sufficiently standardized or precise manner to allow for a valid comparison between the control and intervention arms. For this reason, an area-based comparison could not be included in this study. Instead, we relied on the number and proportion of treated habitats to demonstrate reductions in larvicide use. We have clarified this limitation in the revised text and also note that future implementations could benefit from more systematic recording of habitat size to further strengthen such analyses.

***7 Please state why no ethical permission was obtained for drone based surveillance of breeding habitats. Whether the grid used in drone mappring (300x300m) area did not involve any human or cattle habitation. Further, state about ethical clearance for stakeholder and field worker Qualitative insights.***

**Response:**

Thank you for raising this important point. We have clarified these aspects in the revised Methods section under a new subsection “Ethical approval and permissions.” The study protocol was reviewed and approved by the National Malaria Elimination Program (NMEP) under the Ministry of Health of Ghana, which issued directives to the district health office and its sub-health centers. Additional approval and support were obtained from community leaders (e.g., Assembly men) prior to field implementation. All drone flights were conducted in compliance with Ghana Civil Aviation Authority (GCAA) regulations. Mapping covered entire communities, and while incidental images of humans or livestock may have been captured, these were neither analyzed nor used for any purpose; image review was strictly limited to surface waterbody identification. Feedback from field staff and stakeholders was obtained voluntarily during routine operational activities, without collecting personal identifiers. As such, this did not constitute human subjects research requiring separate institutional review board approval. We believe these clarifications address the reviewer’s concern and ensure transparency regarding ethical oversight.

***8 Why traps were placed at one site only in each conventional and interventional subdistricts. Traps placement in all the study location might provide better assessment of entomological outputs.***

**Response:**

Thank you for this comment. In our study, the mosquito sampling unit of observation was defined as the community. Two communities with similar ecology and climate were selected as intervention and control sites. Each community (both conventional and intervention) was divided into four quadrats, and one trap was deployed per quadrat. This ensured spatial coverage across the study area and avoided concentrating all sampling effort in a single location.

We also acknowledge that resource constraints limited our ability to deploy traps across all subdistricts. Therefore, we based trap placement on subdistricts with prior experience in entomological surveillance, which allowed for more reliable implementation and ensured comparability between the intervention and control sites. While not exhaustive, this quadrat-based design captured spatial heterogeneity within the study areas and provided representative entomological data to support robust comparison.

***9 No statistical tests are presented to compare outcomes between groups, limiting confidence in the reported differences. The study would benefit from formal statistical testing to compare larvicide use, person-days, and mosquito abundance between groups. Present p-values or confidence intervals where appropriate to support claims of efficiency.***

**Response:**

We thank the reviewer for this important comment. In response, we have incorporated formal statistical analyses into the manuscript.

**Methods:** We now state that operational efficiency outcomes were compared between intervention and control groups using the Mann–Whitney U test, given the small sample size and non-normal distribution of the data. The entomological data (species distributions) were analyzed using chi-square tests (with Fisher’s exact test considered where expected cell counts were low).

**Results:** Statistical comparisons showed that the number of person-days per unit area was significantly lower in the intervention sites (median 3.92 [IQR 2.95–4.97]) compared to the control sites (7.23 [IQR 6.39–8.87], Mann–Whitney U = 0.0, p = 0.029). Similarly, the number of sites treated per pack of larvicide was significantly higher in the intervention sites (174 [IQR 164–179]) than in the control sites (49 [IQR 45–64], Mann–Whitney U = 16.0, p = 0.029). In addition, chi-square analysis demonstrated a significant difference in mosquito species distribution between intervention and control areas (χ²(2) = 33.63, p < 0.001), with Anopheles and Aedes being less prevalent in the intervention site (Abaam) and Culex predominating.

**Discussion:** We revised the text to emphasize that these statistical findings support improved operational efficiency in the intervention areas. At the same time, we note the limitation of small sample size (n = 4 per group), and therefore interpret the results as supportive rather than definitive.

We believe these revisions directly address the reviewer’s concern by providing formal statistical testing, reporting p-values, and integrating the findings into the interpretation of operational efficiency and entomological outcomes.

***10 Line 459-484 seems not the part of results. It may be moved to discussion.***

**Response:**

Thank you for this helpful comment. We agree that the epidemiological results in the original manuscript were described in a way that partially overlapped with discussion and could be clarified. In the revised version, we have separated and streamlined the Results and Discussion sections. Specifically, in the Discussion, we now explicitly acknowledge the limitations of interpreting epidemiological outcomes. We highlight that the four-month intervention window may have been too short to detect conclusive effects, that the intervention period overlapped with the natural seasonal decline in malaria transmission, and that other confounding factors such as meteorological variation, concurrent interventions, and reporting differences may have influenced case counts. Taken together, we note that the observed reductions are more plausibly explained by seasonal variation rather than the intervention itself. Importantly, we emphasize that outpatient malaria outcomes were not inferior despite reduced larvicide use and labor inputs, underscoring the operational value of the proposed approach. We believe this restructuring improves clarity and ensures that the Results section presents only observed findings, while interpretation and limitations are confined to the Discussion.

***11 There is some inconsistency in results. Kindly present in clear and concise way. Specially line 533-594.***

**Response:**

Thank you for this helpful comment. We agree that the section originally spanning lines 533–594 contained overlapping descriptions that could reduce clarity and create the appearance of inconsistency.

In the revised manuscript, we have streamlined this section to ensure that the Results present only observed findings, while interpretation and contextualization are placed in the Discussion. We have also clarified that the perspectives of clinicians and field staff represent subjective impressions rather than systematically collected quantitative data. This restructuring improves consistency across sections and provides a clearer narrative distinction between empirical findings and interpretive insights.

We believe these changes address your concern and enhance the readability and accuracy of the results.

***12 In discussion authors suggest mapping by drone and spraying manually. Both mapping and spraying by AI enabled drone technology could result in better output.***

**Response:**

Thank you for this thoughtful suggestion. We agree that incorporating spraying drones represents a promising direction toward more automated LSM. In the revised Discussion, we have added a section noting that while our study focused on combining drone-based mapping with manual spraying, future integration of spraying drones could further enhance automation. At the same time, we emphasize that integrating large and heavy spraying drones into urban environments while ensuring safe operation remains a critical challenge. We note that future research should evaluate the feasibility of spraying drones under appropriate ecological and operational conditions, with particular attention to safety and community acceptance. This revision complements our discussion of future integration of lightweight computer vision models for waterbody detection, together highlighting pathways toward greater scalability and sustainability of digital LSM.

***13 In methodology, there is no details of Feedback from field staff.***

**Response:**

Thank you for this helpful comment. We agree that details on feedback from field staff were missing. As suggested, we have now added a description in the Methods section to clarify that research members accompanied the spraying activities, engaged in direct communication with field staff, and later organized a validation workshop to gather stakeholder feedback.

***14 Kindly provide quantitative results for Qualitative insights from Local Stakeholders. How many field and stakeholder feedbacks were obtained. How many of those reported what.***

**Response:**

Thank you for this helpful comment. We agree that quantitative results would strengthen the qualitative insights from local stakeholders. However, in this pilot study we did not conduct structured surveys or systematically record the number and attributes of participants providing feedback. As such, it is not possible to present quantitative breakdowns of stakeholder responses. We have acknowledged this as a limitation in the Discussion, noting that these insights represent subjective perceptions rather than systematically collected data, and that future studies should incorporate structured surveys or mixed-methods evaluations to capture stakeholder and community feedback alongside quantitative outcomes.

***15 In introduction, details of malaria cases of 2023 may be provided.***

**Response:**

Thank you for this suggestion. We have updated the Introduction section to incorporate the most recent data from the World Malaria Report 2024. Specifically, we now note that in 2023 the global malaria burden further increased to an estimated 263 million cases and 597,000 deaths, with sub-Saharan Africa accounting for 94% of cases and 95% of deaths. We believe this addition strengthens the contextual framing of our study.

***16 Legends for figure may be defined in better way.***

**Response:**

Thank you for this comment. We have revised and clarified the legends of all figures. Where necessary, we expanded abbreviations, added units, and supplemented explanations of groups or categories. We believe these changes make the figures more self-explanatory and easier to understand.

**・Response to Reviewer #2:**

***1 Here the larvicide was Vectolex WDG which is Bacillius spharicus 2362 (strain ABTS - 1743) was used. I do not fine its efficacy. Because its efficacy depends on exposure to sunlight, pH of water and in fragile ecosystem. Please give details.***

**Response:**

Thank you for this comment. We have revised the Methods section to clarify the larvicide used in our study. Both the conventional and proposed LSM approaches employed VectoLex WDG, a biological larvicide containing Bacillus sphaericus 2362 (strain ABTS-1743), with a reported residual efficacy of 3–4 weeks. VectoLex is formulated as a water-dispersible granule and can be applied with standard ground or aerial equipment across diverse aquatic habitats. In Ghana, larvicide selection and application protocols are determined by the National Malaria Elimination Program (NMEP), under which VectoLex has been adopted as the standard product for operational LSM activities. Our study therefore followed existing national policy, with the primary focus placed on evaluating the feasibility of drone- and AI-assisted mapping rather than reassessing the baseline efficacy of the larvicide.

***2 Economic validation.***

**Response:**

Thank you for this valuable comment. In our study, we assessed the economic implications of the proposed approach by comparing labor requirements and the number of habitats treated per larvicide pack. We did not perform a detailed monetary cost analysis because unit labor costs vary significantly across countries and specific larvicide procurement costs were not available due to confidentiality agreements between the NMEP and suppliers. Presenting results in these operational terms also allows readers to adapt the findings to their own local cost structures. At the same time, we recognize the importance of providing more concrete insights. Therefore, we have added in the Results section an illustrative calculation estimating how many workdays of labor savings would be required to offset the initial cost of drone procurement.

***3 Community acceptance.***

**Response:**

Thank you for this important comment. Community acceptance of drone flights was not formally assessed in this study. However, no adverse incidents or resistance were reported during drone operations, and we have added a description in the Discussion summarizing the observed community reactions during the trial. For example, in some communities residents were welcoming and even assisted spray teams, while in others limited sensitization led to some hesitation regarding larvicide application or concerns about drone flights. Future work should therefore include systematic evaluation of community perceptions and acceptance, as these are critical for the sustainability and scalability of LSM interventions.

We have added clarification in the Methods section regarding drone flight permissions and community engagement procedures.

***4 Impact on other insects.***

**Response:**

We appreciate this important point. In the revised Methods section, we have added clarification that VectoLex WDG is highly specific to mosquito larvae, minimizing impacts on non-target aquatic organisms such as fish and beneficial invertebrates. This selectivity contributes to its environmental safety and suitability for operational LSM programs, even in fragile ecosystems.

***5 Larval data missing.***

**Response:**

Thank you for this comment. We acknowledge that systematic larval surveys were not conducted due to resource constraints, as the study was implemented as an operational extension of the National Malaria Elimination Program (NMEP). However, to support the evaluation of the Larval AI, larval presence was assessed in a subset of intervention areas during operations in November. Beyond this limited effort, intervention outcomes were primarily evaluated through changes in adult mosquito abundance and outpatient malaria case records (OPD data). We have added this clarification as a limitation in the revised Discussion.

***6 N/A***

***7 Most mosquitoes were Culex. Impact of any diseases transmitted by Culex spp.***

**Response:**

Thank you for this comment. Our study was designed to evaluate malaria control through reduction of Anopheles mosquitoes, but Culex mosquitoes were predominant in our collections, which we acknowledge as a limitation of the study. While Culex species are globally recognized vectors of diseases such as West Nile virus, Japanese encephalitis, and lymphatic filariasis, they are not known to transmit these pathogens in Ghana [33]. Therefore, in the local context, their reduction does not directly contribute to malaria or other disease transmission control. Nevertheless, reducing Culex populations has important ancillary benefits, including the prevention of biting nuisance and improvement of community well-being, as people sleep better and are more productive when they are not disturbed by mosquito bites and noise. We have added this clarification in the revised Discussion.

***8 Limitations such as inability of breeding detection during dry season and also breeding habitats covered under canopy.***

**Response:**

Thank you for this important comment.

We address your concern as follows and have also updated the Discussion section to reflect this point.

1 Study period and climate context

The study was conducted from September to December in the Eastern Region of Ghana, a period classified largely as dry season (with September marking the transition from rainy to dry).

2 Evidence from our findings

Despite being carried out mostly in the dry season, drone surveys still detected an increased number of waterbodies. This indicates that the approach is effective even under dry-season conditions, and its effectiveness would likely be further enhanced during the rainy season.

3 Recognized limitations

We acknowledge that adverse weather conditions (e.g., heavy rain, strong winds) may restrict drone flights, and that breeding sites completely hidden under canopy or buildings cannot be detected from the air.

4 Mitigation and operational implications

Importantly, the method enables clear identification of areas where drone mapping is effective and where it is not, allowing complementary allocation of ground-based surveys. This combined approach could improve overall operational efficiency.

***9 Whether other vector control agents such as larvivorous fish can be used?***

**Response:**

Thank you for this comment. We agree that biological control agents such as larvivorous fish represent alternative approaches within larval source management. In response, we have briefly acknowledged these strategies in the Introduction as part of the broader range of LSM strategies, while keeping the focus of our study on improving the efficiency of larvicide-based interventions.

**・Response to Additional Editor’s Requirements:**

**Additional Editor Comments:**

**Although the authors have tried to answer most of the comments by reviewer 1, a few things regarding LSM should be further expanded upon, such as economic value and community acceptance as that adds dimensions to vector control.**

**Response:**

We sincerely thank the Academic Editor for the constructive additional comments. In response to the request to further expand on aspects of larval source management (LSM), particularly the economic value and community acceptance, we have revised the manuscript to strengthen these dimensions and provide a fuller contextual understanding of the proposed drone- and AI-assisted workflow.

1. Economic value

To address the Editor’s comment, we have added a detailed discussion on the cost-related implications of implementing drone- and AI-assisted LSM. Specifically, in the Results section, we clarified the potential cost-effectiveness of the proposed approach. The newly added text explains that the workflow can reduce labor requirements by approximately half, aligning with prior evidence indicating that labor constitutes around 58% of LSM operational costs. We also describe how these reductions can improve scalability under fixed budgets, how initial drone investments may be recovered within roughly one year, and how improvements in spraying efficiency may decrease larvicide consumption. The added paragraph in discussion is as follows:

*From an economic perspective, the findings of this study indicate that the introduction of drones and AI can potentially reduce labor costs by approximately half, even when implementing LSM at scales comparable to conventional methods. Previous study on LSM cost structures have reported that labor accounts for about 58 percent of total costs [43], suggesting that the proposed approach could substantially alleviate this major cost component. The magnitude of labor cost savings is expected to increase as the operational area expands, which implies that larger LSM coverage may be achievable within the same budget. In addition, for operations of a scale like that examined here, the initial investment required for drone procurement could be recovered within roughly one year through labor cost savings alone, indicating a favorable cost-effectiveness profile over the long term. Larvicide constitutes another considerable portion of LSM costs, accounting for around 14 percent [43]. Improvements in spraying efficiency can therefore reduce larvicide consumption, leading to lower operational costs per cycle. Overall, the proposed method may enable LSM implementation with fewer personnel and reduced larvicide use while maintaining or lowering total budget requirements, making it a promising strategy for sustainable vector control in resource-constrained settings.*

2. Community acceptance

We also expanded the Discussion to include a fuller description of community engagement, resident perceptions, and operational safety considerations. We highlight the steps taken to ensure transparency and local cooperation, resident concerns regarding noise, crash risk, and privacy, and mitigation strategies implemented during field operations. We also contextualize drone noise levels relative to WHO guidelines and clarify how privacy was protected under the national regulatory framework. The added paragraph in discussion is as follows:

*To support community understanding and cooperation, community engagement activities were conducted in each sub-district prior to implementation. Explanatory meetings with community elders were held to secure approval for the purpose, procedures, and flight plan of the LSM operation, and additional announcements were disseminated through local public speaker systems. Information sharing continued throughout the intervention period with support from the district NMEP office. Although some residents expressed hesitation or unease, no conflicts or operational disturbances were reported, and LSM activities proceeded smoothly, likely reflecting both prior community experience with LSM interventions and the effectiveness of early communication efforts.*

*Resident concerns regarding drone use centered on noise, crash risk, and privacy. With respect to noise, the sound of multirotor propellers may be perceived as unpleasant. However, the Mavic 3M was operated at approximately 100 m altitude, which substantially reduced sound pressure at ground level. Previous studies report that drones of similar size generate approximately 40 to 50 dB directly beneath the aircraft at about 120 m altitude [43]. These values fall within or below the World Health Organization daytime guideline thresholds for transportation noise, which are 53 dB for road traffic and 45 dB for aircraft [44]. Consistent with these expectations, no noise-related complaints were recorded. Even so, thresholds for noise annoyance vary across individuals and cultural contexts, indicating the need for future work that incorporates acoustic measurements and resident surveys. Crash risk cannot be eliminated entirely, but several mitigation measures were implemented. These included mandatory helmet use for field teams, restricting flights to an area of 300 m by 300 m that could be visually monitored, operating only with fully charged batteries, and avoiding adverse weather conditions. Regarding privacy, flight permission was obtained under regulations that prohibit secondary use of collected imagery. At 100 m altitude, the ground sampling distance was approximately 3 cm per pixel, which allows recognition of human presence but makes personal identification highly unlikely. Although household structures were visible, which introduces a residual risk of inferring household characteristics, such information can support malaria control activities such as planning for indoor residual spraying. Maintaining an appropriate balance between public health utility and privacy protection remains essential.*

We believe these additions substantially enhance the manuscript by addressing the broader operational and social dimensions of LSM implementation, as requested. We sincerely appreciate the Editor’s guidance and hope that the revisions now meet the journal’s requirements.
